# A Perspective on Pediatric Respiratory Outcomes During California Wildfires Due to Smoke and PM_2.5_ Exposure

**DOI:** 10.3389/fped.2022.891616

**Published:** 2022-07-06

**Authors:** Sarah M. Naughten, Rosana Aguilera, Alexander Gershunov, Tarik Benmarhnia, Sydney Leibel

**Affiliations:** ^1^Division of Biological Sciences, University of California, San Diego, San Diego, CA, United States; ^2^Scripps Institution of Oceanography, University of California, San Diego, San Diego, CA, United States; ^3^Division of Allergy, Immunology, and Rheumatology, Department of Pediatrics, and Herbert Wertheim School of Public Health and Human Longevity Science, University of California, San Diego, San Diego, CA, United States; ^4^Rady Children's Hospital, San Diego, CA, United States; ^5^Herbert Wertheim School of Public Health and Human Longevity Science, University of California, San Diego, San Diego, CA, United States

**Keywords:** PM_2.5_ exposure, pediatric, respiratory, California wildfires, Air Quality Index (AQI) efficiency

## Abstract

As wildfires increase in prevalence and intensity across California and globally, it is anticipated that more children will be exposed to wildfire smoke, and thus face associated adverse health outcomes. Here, we provide a concise summary of the respiratory effects of California's wildfires on pediatric healthcare utilization, examine global examples of wildfire smoke exposure within the pediatric population and associated physiological effects, and assess the efficacy of metrics used to measure and communicate air quality during wildfires within the United States and elsewhere.

## Introduction

Climate change has been increasing wildfire activity in California (1). In 2021 alone, wildfires burned across ~2.6 million acres in California, damaging over 3,000 structures and resulting in three fatalities (2). In addition to such impacts, wildfire smoke severely affects air quality by increasing the concentration of air pollutants. PM_2.5_, airborne particles with a diameter of ≤2.5 μm, are of particular concern since they are small enough to penetrate the deepest recesses of the lungs and enter the bloodstream, thus adversely affecting human health in the immediate surroundings and in areas downwind of wildfires (3). Currently, most studies on the adverse respiratory impact of wildfires focus on adults while fewer specifically consider impacts of wildfire smoke exposure on children's developing lungs (4).

Wildfire smoke is currently a major source of PM_2.5_ pollution in regions like the Western United States (5), and this source of pollution is projected to increase in the context of climate change and variability (6). Wildfire smoke is also a growing concern for public health after studies performed in mice observed that wildfire PM produced more oxidative stress and a greater inflammatory response when compared to other sources of PM (7). Other studies have found that concentrations of gas phase organic compounds and fine particle carbons measured during a wildfire episode were significantly higher than concentrations originating from urban air pollution (8). Although wildfire smoke appears to be more harmful than urban air pollution on human health (9), metrics used to communicate air quality to the public in the US, i.e., the Air Quality Index (AQI; https://www.airnow.gov/aqi/), fail to communicate current knowledge regarding the health impacts associated with PM_2.5_ emitted directly from wildfires (10) nor consider specific vulnerability to such impacts among subgroups such as children. Two gaps in knowledge we have identified concern are: (1) A paucity of short- and long-term respiratory impact data in the pediatric population; (2) How to best communicate air quality related to PM_2.5_ exposure to the public during wildfire episodes.

## Physiological Impacts of Wildfire Smoke on Children

Children are particularly vulnerable to detrimental health impacts of wildfire smoke (11). During childhood respiratory development, lungs are more prone to injury and damage (11). Inhaling chemicals and other irritants, including PM_2.5_, are expected to yield more severe damage in children than in adults, *via* oxidative stress and inflammation (11, 12). In addition, children's smaller airway sizes, when compared to those of adults, raise their sensitivity to airborne pollutants in wildfire smoke by increasing particle deposition in children's lungs (13). Lastly, the increased respiratory rate of children compared to adults increases the dose of wildfire smoke (or any airborne pollutant) inhaled, thus putting children at a greater risk of detrimental respiratory outcomes such as lung damage (11, 14).

Although limited research has been conducted on how exposure to wildfire smoke impacts children's lungs, animal research provides insight into the physiological impact of wildfire smoke exposure on developing lungs. In 2008, a group of researchers in Northern California unintentionally exposed a colony of infant rhesus macaque monkeys to high levels of PM_2.5_ originating from a nearby wildfire. A colony of monkeys born the year after, and thus not exposed to wildfire smoke during infancy, were used by researchers as a control group. After evaluating both groups of monkeys during their adolescence, researchers determined that exposure to wildfire smoke as infants resulted in impaired lung function during adolescence. Although researchers concluded that exposure to wildfire smoke resulted in pulmonary damage that persisted with time, it is unknown whether said damage was detectable immediately following exposure. Animals exposed to wildfire smoke as infants tended to have a lower total lung volume when they reached adolescents (15).

A similar study, examined the consequences of early pregnancy exposure to wildfire smoke from the 2018 Camp Fire within rhesus macaque monkeys (16). The Camp Fire originated in Butte County California and resulted in elevated PM_2.5_ concentrations from November 9 to November 22 2018 (17, 18). The monkeys conceived before or on November 22 2018, were deemed by the researchers to be exposed to high levels of wildfire smoke, including high concentrations of PM_2.5_, during early pregnancy. A colony of monkeys conceived after November 22, 2018, and thus evaded exposure to wildfire smoke, were used by the team as a control group. Capitanio et al. (16) concluded that the animals exposed to wildfire smoke displayed elevations in a marker of inflammation and a varied physiological response to stress.

## Wildfires' Impacts on Pediatric Health Care Utilization

Researchers have been documenting the impact of wildfire PM_2.5_ exposure on health care utilization since the early 2000s. During a series of wildfire events in 2003 in Southern California, Delfino et al. (21) found that asthma admissions in children aged 0–4 years increased by 8.3% per 10 μg/m^3^ increase in PM_2.5_ concentration, while admissions in adults increased by 4.1%. During the post-wildfire period, children aged 0–4 saw a 51% increase in admissions for acute bronchitis and bronchiolitis and a 46% increase in pneumonia admissions (21). Künzli et al. (20) examined the same devastating events in 2003 and observed that during the wildfire episode elementary school-aged children were almost twice as likely to visit a doctor for respiratory symptoms than their high school-aged counterparts.

In another study in Southern California, Hutchinson et al. (3) estimated a mean of 89.1 μg/m^3^ PM_2.5_ concentration in the San Diego area during a series of several wildfires burning in October 2007. During this period, visits to emergency departments (ED) increased significantly for respiratory diagnosis, asthma, upper respiratory infections, acute bronchitis, and other respiratory symptoms for children aged 0–4 (3). Children aged 0–1 had the greatest increase in the presentations of symptoms identified in an ED for respiratory diagnoses (3).

After accessing pediatric health care utilization during the Lilac Fire in 2017, Leibel et al. (4) found that exposure to PM_2.5_ associated with wildfire smoke resulted in an increase in children aged 0–19 seeking medical treatment for respiratory symptoms, with children aged 0–5 reporting the most additional visits per day. While the Lilac Fire was active, the mean daily respiratory visits to pediatric hospital and urgent care facilities, jumped from 55 visits the week before the fire to 75.1 visits during the fire (4). Children aged 6 to 12 years old had an increase of 3.4 daily visits and children aged 0–5 years old had an increase of 7.3 visits per day (4).

Researchers across the globe have determined that wildfire episodes result in increased reports of respiratory symptoms (20, 22, 23, 25). By assessing respiratory symptoms *via* questionnaires, Künzli et al. (20) and Mirabelli et al. (22) determined that increases in wildfire specific PM_10_ resulted in an increase in respiratory symptoms across the pediatric population. Researchers assessing the impact of wildfires in Spain and Chile have also concluded that during wildfire episodes, reports of respiratory symptoms increase across the pediatric population (23, 25).

In addition to increased ED admissions and increased reports of respiratory outcomes, preliminary studies suggest that increase in the concentration of wildfire specific PM, increases the use of albuterol inhalers (an inhaler used to treat asthma (31)); a 2004 study assessing the impact of the 1994 bushfire episode in Sydney, Australia, found that during the brushfire reports of inhaled beta-agonist (another inhaler used for asthma (32)) increased to 13.2 from 9.2% and reports of inhaled corticosteroid use increased to 20.8 from 13.8% (19).

Although researchers have known that wildfire episodes increase pediatric healthcare utilization and respiratory symptoms for decades, recent studies suggest that PM originating from wildfires may be more harmful than PM from non-wildfire sources (9). After assessing pediatric health outcomes in San Diego County from 2011 to 2017, Aguilera et al. (9) determined that non-smoke PM_2.5_ resulted in a 3.7% increase in pediatric respiratory visits, while wildfire specific PM_2.5_ resulted in a 30.0% increase in pediatric respiratory visits. The authors concluded that PM_2.5_ from wildfires can be about 10 times more harmful as PM _2.5_ from non-wildfires sources (9).

While we are unable to identify any studies that investigate the long-term effects of acute PM_2.5_ exposure from wildfire on children's respiratory outcomes, preliminary studies suggest that exposure to smoke specific PM_2.5_ may impair lung function later in life. A 2020 study by Shao et al. concluded that 3 years after the initial exposure to PM_2.5_ from a coal mine fire in 2014 in Australia there remained an association between exposure to high concentrations of PM_2.5_ and impaired lung function. After assessing the lung function of children who were exposed to fire-specific air population while under 2 years of age, the authors detected a small association between PM_2.5_ exposure and impaired lung function (12).

Another study by Shao et al. (13), analyzed the association between PM_2.5_ exposure (from the same coal mine fire above) during infancy and frequency of general practitioner visits and medication use during the year following the fire. The team found that children who were exposed to PM_2.5_ while under 2 years of age displayed increased use of antibiotics when compared to children who were not exposed; the exposed group had an average of 1.5 prescriptions per child per year for antibiotics in the year following the fire, while the non-exposed group had an average of 0.8 prescriptions per child per year (13).

Willis et al. (24) analyzed the long-term effects on health outcomes 2–4 years after the above coal fire episode and concluded that exposure to increased concentrations of PM_2.5_ from the coal fire while *in utero* was associated with impaired respiratory health and increased health care utilization 2–4 years after exposure. Table 1 summarizes the studies assessing pediatric health care outcomes during wildfires.

**Table 1:** Summary of wildfire and PM_2.5_ pediatric respiratory impact studies reviewed (organized chronologically by year published).

**Author**	**Timeframe of study**	**Region studied**	**Breadth of study**	**Key findings**
Jalaludin et al. (19)	January 1994	Sydney, Australia	32 children with a mean age of 9.2 years	The authors found that reports of evening wet cough increased substantially during the brushfire episode (reports of wet coughing increased from 5.5 to 17.0%). Inhaled steroids use also increased substantially during the brushfire period; reports of inhaled beta-agonist use increased to 13.2 from 9.2% and reports of inhaled corticosteroid use increased to 20.8 from 13.8%
Künzli et al. (20)	October 2003	Southern California	Assessed smoke-exposure related symptoms in 873 17–18-year old's and 5,551 6–7-year-olds. Thus, a total of 6,424 participants were included in the study	The authors found that wildfire smoke exposure resulted in an overall increase in respiratory symptoms across all age groups and noted these increase were correlated with concentrations of wildfire-originating PM_10_ Elementary school aged children were almost twice as likely to visit a doctor for respiratory symptoms during the wildfire (11.4% of the elementary-school aged children reported visiting a doctor for respiratory symptoms while 6.7% of the high-school aged children reported visiting a doctor for respiratory symptoms). The elementary-school aged children were also much more likely to use medication to relieve respiratory symptoms than their high-school counterparts
Delfino et al. (21)	October 2003	Southern California	Assessed hospital respiratory admissions throughout southern California	Authors found that per 10 μg/m^3^ increase in PM_2.5_ concentration there was an 8.3% increase in asthma related admissions in children ages 0–4. The same increase in PM_2.5_ resulted in a 4.1% jump in admissions within the adult population (ages 20–64) During the post-wildfire period, there was a 51% increase in admissions related to acute bronchitis and bronchiolitis and a 46% increase in pneumonia admissions, within children ages 0–4
Mirabelli et al. (22)	2003	California	465 nonautomatic children aged 16–19	The authors concluded that wildfire smoke exposure resulted in an increase in the respiratory symptoms evaluated (dry and wet cough, wheezing, and eye irritation). Additionally, increased duration of wildfire episodes resulted in increased prevalence of the respiratory symptoms. This correlation was strongest within children with a diminished lung function ratio. Thus, the authors concluded that within no asthmatic children ages 16–19, small airway size may be indicative of increased vulnerability to effects associated with wildfire smoke exposure
Vicedo-Cabrera et al. (23)	July 2012	Valencia, Spain	460 individuals	The authors concluded that during the 2012 wildfire episode, reports of children experiencing watery/itchy eyes or developing sore throats more than doubled when compared to the non-wildfire control period. Every respiratory symptom assessed reported an increase in prevalence during the wildfire period. The authors also identified children with asthma or rhinitis as an especially vulnerable group
Hutchinson et al. (3)	October 2007	San Diego County, California	Assessed hospital respiratory admissions throughout San Diego County	Authors found that during a period of increased PM_2.5_ concentration, visits to emergency departments for respiratory related conditions, increased significantly for children aged 0–4, with children aged 0–1 exhibiting the greatest increase
Shao et al. (12)	2014	Victoria, Australia	84 children with an average age of 4.3 years	Authors found that exposure to fire specific PM_2.5_ while under 2 years of age was associated with decreased lung function 3 years after said exposure
Shao et al. (12)	2014	Victoria, Australia	a total of 286 children, 77 of which were not exposed, 88 exposed *in utero*, and 121 with exposure while in infancy	Exposure to fire specific PM_2.5_ while under 2 years of age was associated with increased use of antibiotics in the year following exposure
Leibel et al. (4)	December 2017	San Diego County, California	Evaluated emissions to Rady Children's Hospital and University of California clinics throughout San Diego County	Authors found that exposure to wildfire specific PM_2.5_ resulted in increases in respiratory symptoms across the pediatric population, with children ages 0–5 reporting the most additional visits per day
Willis et al. (24)	2014	Victoria, Australia	79 children exposed to smoke while *in utero*, 81 children exposed while under 2 years of age, and 129 children who were not exposed	Exposure to fire specific PM_2.5_ while *in utero* was associated with impaired respiratory health and increased health care utilization 2–4 years after the initial exposure. Additionally, Children exposed to elevated PM_2.5_ levels while under 2 years old displayed a small increase in asthma inhaler use
Aguilera et al. (9)	2011–2017	San Diego County, California	Evaluated emissions to Rady Children's Hospital throughout San Diego County	Authors found that PM_2.5_ from non-wildfire origins resulted in a 3.7% increase in pediatric respiratory visits, while wildfire specific PM_2.5_ resulted in a 30.0% increase. Thus, the authors concluded that PM _2.5_ from wildfires can be about 10 times more harmful as PM _2.5_ from non-wildfires sources
Ciciretti et al. (25)	2010–2013	Santiago and Valparaiso, Chile	Evaluated emission to Emergency Room in Santiago and Valparaiso, Chile	The authors identified that, during the wildfire episode, in Santiago, children under the age of 1 faced increased risk of bronchitis, chronic lower respiratory diseases and pneumonia. Within Santiago, children ages 1–4 also faced increased risk of pneumonia

## Relationship Between PM_2.5_, AQI, and Other Methods of Communicating Air Quality

The standard measure of air quality in the United States, the Air Quality Index (AQI), provides guidance about air pollution to healthcare providers as well as the general public, including schools and parents (33). Based on the AQI during a wildfire, schools and healthcare providers may make decisions affecting children regarding masking and limiting outdoor activities (34). The AQI, however, does not include updated information regarding the impact of PM_2.5_ exposure according to particle chemical composition, sources of emissions nor growing evidence supporting exacerbated impacts among children and subgroups of the population. Specifically, the AQI communicates the dangers associated with six different air pollutants: ozone (O_3_), particle pollution (PM_2.5_ and PM_10_), carbon monoxide (CO), sulfur dioxide (SO_2_), and nitrogen dioxide (NO_2_). When calculating air quality associated with PM_2.5_, the AQI only considers the concentration of PM_2.5_ , disregarding the source of emission of PM_2.5_ (35), i.e., whether pollution is emitted from traffic or industrial activities, or wildfires.

In other regions such as Canada, researchers have raised concerns about the efficacy of standard measurement of air quality (Air Quality Health Index (AQHI)) during wildfire episodes (27). The AQHI is calculated based on the concentrations of O_3_, NO_2_, and PM_2.5_ and conveys air quality *via* a 1–10 scale (with 1 being low risk and 10 being high risk). Because AQHI considers risk associated with O_3_, NO_2_ and PM_2.5_, increased concentrations of PM_2.5_ during wildfire episodes may fail to push AQHI into the moderate/high risk category. Additionally, the AQHI value that is reported each hour is based on a 3-h average of each pollutant considered, and thus may fail to communicate risk associated with rapid increases in PM_2.5_ concentrations during wildfire episodes (26, 36). For instance, as noted by Yao et al. (27) during a 2014 wildfire episode in British Columbia, the highest AQHI value recorded was during a period of high O_3_ concentration. However, during peak PM_2.5_ concentrations, a lower AQHI value was recorded (27).

The AQHI has since been updated to reflect current knowledge regarding the risks of PM_2, 5_ exposure. The region of British Columbia implemented an addendum to the AQHI which ensured that if the PM_2.5_ concentration exceeded 60 μg/m^3^, the *Trump* & *Hold* AQHI-Plus value would be reported as 7, regardless of if the AQHI value was less than 7. The *Trump* & *Hold* AQHI-Plus value would remain as 7 for the following 5 h if the PM_2.5_ concentration was greater than 25 μg/m^3^ during the hour following the initial 60+ μg/m^3^ PM_2.5_ concentration (27).

Over a period of eight wildfire seasons from 2010 to 2017, Yao et al. (27) examined the relative effectiveness of AQHI, *Trump* & *Hold* AQHI-plus and three other metrics, placing particular emphasis on the ability of the evaluations of air quality to predict the prevalence of adverse health impact due to wildfire smoke. The *1-h PM*_2.5_ *Only* AQHI-Plus method of accessing air quality, one of the other metrics evaluated by Yao et al., was developed in order to better communicate the impact that gradual increases in PM_2.5_ has on air quality. Like the *Trump* & *Hold* AQHI-Plus , the *1-h PM*_2.5_ *Only* AQHI-Plus method ensures that when concentration of PM_2.5_ reaches 60 μg/m^3^, the AQHI-plus value will be reported as 7, however, the *1-h PM*_2.5_ *Only* method also ensures that when concentrations of PM_2.5_ are over 30 μg/m^3^ the AQHI-plus value will be reported as 4. Yao et al. (27), concluded that this measurement of air quality was the best predictor of the respiratory-related health impacts, especially for individuals with asthma, during high intensity wildfire episodes. Thus, the authors recommended that the *1-h PM*_2.5_ *Only* AQHI - Plus mechanism be used to communicate air quality during wildfire season (27).

In an effort to combat the negative health outcomes of air pollution exposure, Gladson et al. (29), conducted a study to determine the best mechanism for respiratory risk among children (29). The team found that the most effective communicators of risk globally were indexes that adjusted for extreme pollution values while simultaneously considering the effect of co-pollutants (29).

In addition to updating the metric used to evaluate air quality, researchers have noted that smoke-forecasting systems and other prediction systems may aid in implementation of improved public health responses. A 2013 study by Yao et al. (26) assessed the utility of one such system, the BlueSky Western Canada Wildfire Smoke Forecasting Framework. This technology has provided the public with forecasts of PM_2.5_ concentrations up to 60 h in advance since 2010. After evaluating the technology's performance for a period of 35 days during fire season (from July 24 to August 29, 2010), the team concluded that there was modest agreement between the PM_2.5_ concentration predicted by BlueSky technology and the hourly measured PM_2.5_ concentrations (26). The authors also concluded that each 30 μg/m^3^ increase in BlueSky's PM_2.5_ prediction was correlated with an 8% increase in salbutamol (a common asthma prescription) dispensations and 5% increase in asthma-related physician visits (26, 31).

The efficacy of another prediction system, the British Columbia Asthma Prediction System (BCAPS), was assessed by Henderson et al. (30) during British Columbia's 2018 wildfire season. The BCAPS uses historical data on PM_2.5_ concentrations and subsequent health outcomes to predict the impact of PM_2.5_ exposure in the upcoming days. The inhaler dispensation forecasts made by the BCAPS during the study period were within 20% of the actual value in 71% of observed cases. The authors noted that BCAPS provided users with valuable insights regarding the impact of expected smoke exposure (30).

Within Australia, another modeling technique, Air Quality Visualization (AQVx), was trialed in Victoria, a region of south-eastern Australia plagued by both frequent wildfire episodes during the summer and large-scale prescribed burnings (28). The AQVx system incorporates air quality metrics, satellite imagery, radar, and crowd-sourced information (health symptoms, smoke sightings, etc.) into the final interface. According to Williamson et al. (28), the AQVx system provided the Department of Environmental, Land, Water, and Planning with information necessary to identify populations at risk of smoke exposure. Table 2 Summarizes the studies assessing efficacy of communicating air quality.

**Table 2:** Summary of air quality communication studies reviewed (organized chronologically by year published).

**Author**	**Air quality metric analyzed**	**Key findings**
Yao et al. (26)	BlueSky Western Canada Wildfire Smoke Forecasting Framework	Authors concluded that there was modest agreement between BlueSky's PM_2.5_ prediction and the measured PM_2.5_ concentration
Yao et al. (27)	AQHI, *Trump & Hold* AQHI-plus and three other metrics	Authors found that the *1-h PM_2.5_ Only* method of accessing air quality was the best predictor of respiratory-related health impact during high intensity wildfire episodes and thus reckoned the *1-h PM_2.5_ Only* AQHI - Plus mechanism be used to measure air quality during wildfire episodes
Williamson et al. (28)	Air Quality Visualization (AQVx)	The authors concluded that the AQVx system aided government agencies in identifying populations at risk for smoke exposure.
Gladson et al. (29)	Analyzed findings of 75 health studies and 32 systematic reviews	Determined that the air quality indexes that most effectively conveyed respiratory risk among children were those that both adjusted for extreme pollution value and communicated dangers of co-pollutants
Henderson et al. (30)	British Columbia Asthma Prediction System	The BCAPS system's inhaler dispensation forecasts were within 20% of the recorded value 71% of the time

## Discussion

As climate change continues to promote hotter and drier conditions in California, exacerbating California's already notorious wildfire seasons (37), more children are expected to be exposed to and disproportionately affected by wildfire smoke (38). Within this paper, our group provided a summary of the existing knowledge regarding both short- and long-term exposure to smoke-specific PM_2.5_ and assessed the efficacy of air quality metrics during wildfire episodes.

Based on the above findings, we are concerned that the respiratory impacts of wildfire specific PM_2.5_ on children is currently being underestimated and needs to be investigated further regarding both short- and long-term health outcomes. Additionally, young children may be particularly at risk for increased respiratory healthcare utilization (4) because of physiologic aspects of lung development. Furthermore, PM_2.5_ from wildfire exposure may have more of a respiratory health impact than ambient PM_2.5_ as suggested by Aguilera et al. (9).

In order to protect children - our most vulnerable population - more specific analysis on the pediatric respiratory effects of PM_2.5_ during wildfires and bolder steps to incorporate the effects of wildfire PM_2.5_ into the USA's AQI, particularly in wildfire prone areas, are both needed.

## Data Availability Statement

The original contributions presented in the study are included in the article/supplementary material, further inquiries can be directed to the corresponding author.

## Author Contributions

SN complied sources and wrote drafts of the manuscript. RA, AG, TB, and SL suggested sources to include and provided SN with comments on drafts. SL provided additional guidance to SN. All authors contributed to the article and approved the submitted version.

## Conflict of Interest

The authors declare that the research was conducted in the absence of any commercial or financial relationships that could be construed as a potential conflict of interest.

## Publisher's Note

All claims expressed in this article are solely those of the authors and do not necessarily represent those of their affiliated organizations, or those of the publisher, the editors and the reviewers. Any product that may be evaluated in this article, or claim that may be made by its manufacturer, is not guaranteed or endorsed by the publisher.
